# Reduction of relative centrifugal forces increases growth factor release within solid platelet-rich-fibrin (PRF)-based matrices: a proof of concept of LSCC (low speed centrifugation concept)

**DOI:** 10.1007/s00068-017-0785-7

**Published:** 2017-03-21

**Authors:** K. El Bagdadi, A. Kubesch, X. Yu, S. Al-Maawi, A. Orlowska, A. Dias, P. Booms, E. Dohle, R. Sader, C. J. Kirkpatrick, J. Choukroun, S. Ghanaati

**Affiliations:** 1 FORM (Frankfurt Orofacial Regenerative Medicine) Lab, Department for Oral, Cranio-Maxillofacial and Facial Plastic Surgery, University Hospital Frankfurt Goethe University, Theodor-Stern-Kai 7, 60590 Frankfurt am Main, Germany; 2 Department of Orthopedics, West China Hospital/West China School of Medicine, Sichuan University, Chengdu, Sichuan People’s Republic of China; 3 Private Practice, Pain Therapy Center, Nice, France

**Keywords:** Inflammation, Leukocytes, Platelets, Platelet-rich-fibrin, Tissue engineering, Vascularization

## Abstract

*Purpose* The present study evaluated the platelet distribution pattern and growth factor release (VEGF, TGF-β1 and EGF) within three PRF (platelet-rich-fibrin) matrices (PRF, A-PRF and A-PRF+) that were prepared using different relative centrifugation forces (RCF) and centrifugation times. *Materials and methods* immunohistochemistry was conducted to assess the platelet distribution pattern within three PRF matrices. The growth factor release was measured over 10 days using ELISA. *Results* The VEGF protein content showed the highest release on day 7; A-PRF+ showed a significantly higher rate than A-PRF and PRF. The accumulated release on day 10 was significantly higher in A-PRF+ compared with A-PRF and PRF. TGF-β1 release in A-PRF and A-PRF+ showed significantly higher values on days 7 and 10 compared with PRF. EGF release revealed a maximum at 24 h in all groups. Toward the end of the study, A-PRF+ demonstrated significantly higher EGF release than PRF. The accumulated growth factor releases of TGF-β1 and EGF on day 10 were significantly higher in A-PRF+ and A-PRF than in PRF. Moreover, platelets were located homogenously throughout the matrix in the A-PRF and A-PRF+ groups, whereas platelets in PRF were primarily observed within the lower portion. ​*Discussion* the present results show an increase growthfactor release by decreased RCF. However, further studies must be conducted to examine the extent to which enhancing the amount and the rate of released growth factors influence wound healing and biomaterial-based tissue regeneration. ​*Conclusion* These outcomes accentuate the fact that with a reduction of RCF according to the previously LSCC (described low speed centrifugation concept), growth factor release can be increased in leukocytes and platelets within the solid PRF matrices.

**Electronic supplementary material:**

The online version of this article (doi:10.1007/s00068-017-0785-7) contains supplementary material, which is available to authorized users.

## Introduction

Various blood concentrates are used to support tissue regeneration and wound healing in different fields. One of these systems is platelet-rich plasma (PRP), a technique that has been developed for clinical practice and tissue regeneration therapies [1, 2]. PRP is prepared by multiple centrifugation steps using patient blood to which anticoagulants have been added to achieve a platelet-rich concentrate that can be used for different indications [3]. However, seeking to minimize contamination risk, eliminate additional anticoagulants and use the autologous and natural regeneration capacity, a new system, platelet-rich fibrin (PRF), was introduced as the first blood concentrate system without additional anticoagulants [4].

PRF is derived from patient venous blood by means of single-step centrifugation without the further addition of any type of anticoagulants. This system was developed to fulfill clinical needs by being time-saving and easy to use [4]. PRF-based matrices include various inflammatory cells, such as platelets and leukocytes, in combination with various plasma proteins embedded in a fibrin network [5]. The components of PRF-based matrices are known to play an important role during the process of wound healing. Platelets are the first cells to occur in the region of an injury. In addition to their role within hemostasis, platelets have inflammatory potential, including the recruitment of further inflammatory cells, such as neutrophils and macrophages, and promote angiogenesis and tissue repair [6, 7]. In this context, platelets are able to express a series of biologically active signaling molecules and growth factors, such as platelet-derived growth factor (PDGF), vascular endothelial growth factor (VEGF) and transforming growth factor beta (TGF-β). These growth factors are essential for tissue vascularization and new tissue formation [8, 9]. Moreover, platelets contain granules with cytokines, chemokines and other inflammatory mediators that are released after platelet aggregation to enhance hemostasis and activate and recruit cells to the site of inflammation [10, 11]. Leukocytes also contribute to angiogenesis and lymphangiogenesis by participating in cell–cell cross talk and expressing various signaling molecules [12, 13]. The extracellular matrix in the wound bed supports the formation of blood vessels, and fibrin provides a scaffold for the inflammatory cells [14].

The structure and constituents of PRF-based matrices were previously explored by our group. An ex vivo histomorphometrical study showed a dense structure and specific localization of the included inflammatory cells in the lower part of PRF [5]. In addition, a modification of the preparation setting based on the previously LSCC (described low-speed centrifugation concept) is a first step in the reduction of the applied relative centrifugation force (RCF). This step was accompanied by a mild increase of centrifugation time, resulting in a so-called advanced PRF (A-PRF) [5, 15]. Analysis of the structure and composition of A-PRF revealed a more porous structure compared to PRF [5]. In addition, histomorphometrical analysis revealed significantly more neutrophilic granulocytes in the group of A-PRF compared with PRF [5].

While developing PRF-based matrices, the focus was on clot formation, consistency and functional integrity the fibrin clot and the distribution of the included inflammatory cells to generate PRF-based matrices with high functionality and adequate handling. In this study, the applied RCF and centrifugation times are key elements. Further research on PRF-based matrices regarding their structure and composition indicates that adjusting the centrifugation time, i.e., reducing the spinning time and applying the same RCF as in the case of A-PRF, allows the introduction of a new PRF-based matrix, Advanced-PRF+ (A-PRF+). A previous systematic study demonstrated the influence of the RCF reduction on the leukocyte and platelet numbers as well as their role in growth factor release in fluid PRF-based matrices following the LSCC, which indicates that reducing the RCF enhances the cell number and growth factor release within PRF-based matrices [15]. Based on the LSCC, we examined modifications of the RCF and centrifugation times in solid PRF-based matricesand their influence on the growth factor release within the previously introduced PRF protocols with a solid structure; PRF, A-PRF and A-PRF+. Therefore, the goal of the present study was to determine growth factor release in solid PRF-based matrices, PRF, A-PRF and A-PRF+, at six different time points over a period of 10 days. Additionally, immunohistochemical analysis was conducted to assess the platelet distribution pattern within the various PRF-based matrices.

## Materials and methods

### PRF preparation

For each protocol, peripheral blood was drawn from four healthy volunteers between 25 and 60 years of age (two females, two males) without a history of anticoagulant usage. Informed consent was obtained from each donor who participated in this study. As previously described [5], the venous blood was collected in 10-ml sterile glass tubes (A-PRF tubes Process for PRF™, Nice, France; Mectron, Cologne, Germany) without external anticoagulants and placed immediately in a centrifuge (Duo centrifuge, Process for PRF™, Nice, France; Mectron, Cologne, Germany). The centrifuge has a fixed angle rotor with a radius of 110 mm and no brake. After centrifugation time, the centrifugation process ends automatically, and the centrifuge stops in 2–5 s. All preparation steps were performed at room temperature according to the established protocols as follows:

- PRF: 10 ml; 2400 rpm; 12 min; 708 g
- A-PRF: 10 ml; 1300 rpm; 14 min; 208 g
- A-PRF+: 10 ml; 1300 rpm; 8 min; 208 g

After centrifugation, all clots were carefully removed from the tubes and separated from the red blood cell fraction with sterile tweezers and scissors.

### PRF cultivation

The total clots of PRF, A-PRF and A-PRF+ were placed in separate wells of a 6-well plate (Greiner, Bio-One International) and covered with 5 ml Roswell Park Memorial Institute medium (RPMI 1640, Gibco Thermo Fischer Scientific) without Fetal Bovine Serum and supplemented with L-glutamine and 1% penicillin/streptomycin. The clots were incubated in a humidified incubator for up to 10 days at 37 °C with 5% CO_2_. The supernatants from each well were taken after 6, 24, 48, 72 h, 7 and 10 days and stored as aliquots at −80 °C. At each time point, all of the clots of PRF-based matrices were placed into new wells and covered with 5 ml fresh medium.

### Growth factor measurement

The supernatants that were collected from the various PRF-based matrices at different cultivation time points were used for the quantification of different growth factors by enzyme-linked immunosorbent assay (ELISA). All collected supernatants were simultaneously centrifuged (1500 rpm; 5 min.) using a centrifuge (Thermo fisher scientific, Heraeus^®^ Labofuge^®^ 400 R) to exclude possible residue that could affect the photometrical measurement. Before TGF-β1 and EGF ELISA preparation, the supernatants were diluted 1:4 with the same cell culture RPMI medium used for PRF-matrices cultivation. The protein concentrations of human VEGF, TGF-β1 and EGF were determined by the Dou Set ELISA kit (Human VEGF DY293B, R&D Systems, detection range: 2000–31.3 pg/ml), HumanDou Set ELISA kit (Human TGF-β1 DY240, R&D Systems, detection range: 2000–31.3 pg/ml) and the Duo Set DuoSet ELISA kit (human EGF DY236, R&D Systems, detection range: 3.91–250 pg/mL) according to the manufacturer´s instructions. Measurements were conducted using a microplate reader (Infinite^®^ M200, Tecan, Grödig, Austria) set to 450 nm and subtracted at 570 nm from the 450 nm measurements.

### Immunohistological analysis

As previously described [5, 16], the PRF clots were collected after 10 days and fixed in Roti^®^*-*Histofix 4%, acid free (pH 7), and 4% phosphate-buffered formaldehyde solution (Carl-Roth) for 24 h. The PRF-based matrices were dehydrated in a series of alcohol and xylene through a Tissue Processor (TP1020, Leica Biosystems Nussloch GmbH, Germany) and embedded in paraffin blocks. Afterwards, 3 µm thick sections from each sample were cut by a rotatory microtome (Leica RM2255, Wetzlar, Germany). For immunohistochemistry, the sections were deparaffinized, rehydrated and finally sonicated in citrate buffer (pH 6) at 96 °C for 20 min. The sections were stained with monoclonal mouse anti-human CD61 marker (1:50, Platelet Glycoprotein IIIa/APC, Clone Y2/5, Dako) by means of an autostainer (Lab vision Autostainer 360, Thermo Fisher Scientific). Histological examination was conducted using a light microscope (Nikon Eclipse 80i, Tokyo, Japan). Three of the authors KE, SA and SG, were independently blinded for the morphological analysis. The microphotographs were prepared with a connected DS-Fi1/Digital camera (Nikon, Tokyo, Japan) and a Digital sight unit DS-L2 (Nikon, Tokyo, Japan).

### Statistical evaluation

Data were expressed as the mean ± standard deviation. Statistical analysis was conducted using Prism Version 6 (GraphPad Software Inc., La Jolla, USA). The significance of differences among means of data was analyzed using two-way analysis of variance (ANOVA) with the Tukey multiple comparisons test (*α* = 0.05) of all pairs. The significant differences were regarded as significant if the *p* values were less than 0.05 (*p < 0.05) and highly significant if the *p* values were less than 0.005 (**p < 0.005), 0.0005 (***p < 0.0005) or 0.0001 (****p < 0.0001).

## Results

### General observation of fibrin clotting within the three investigated groups

**Fig. 1:**
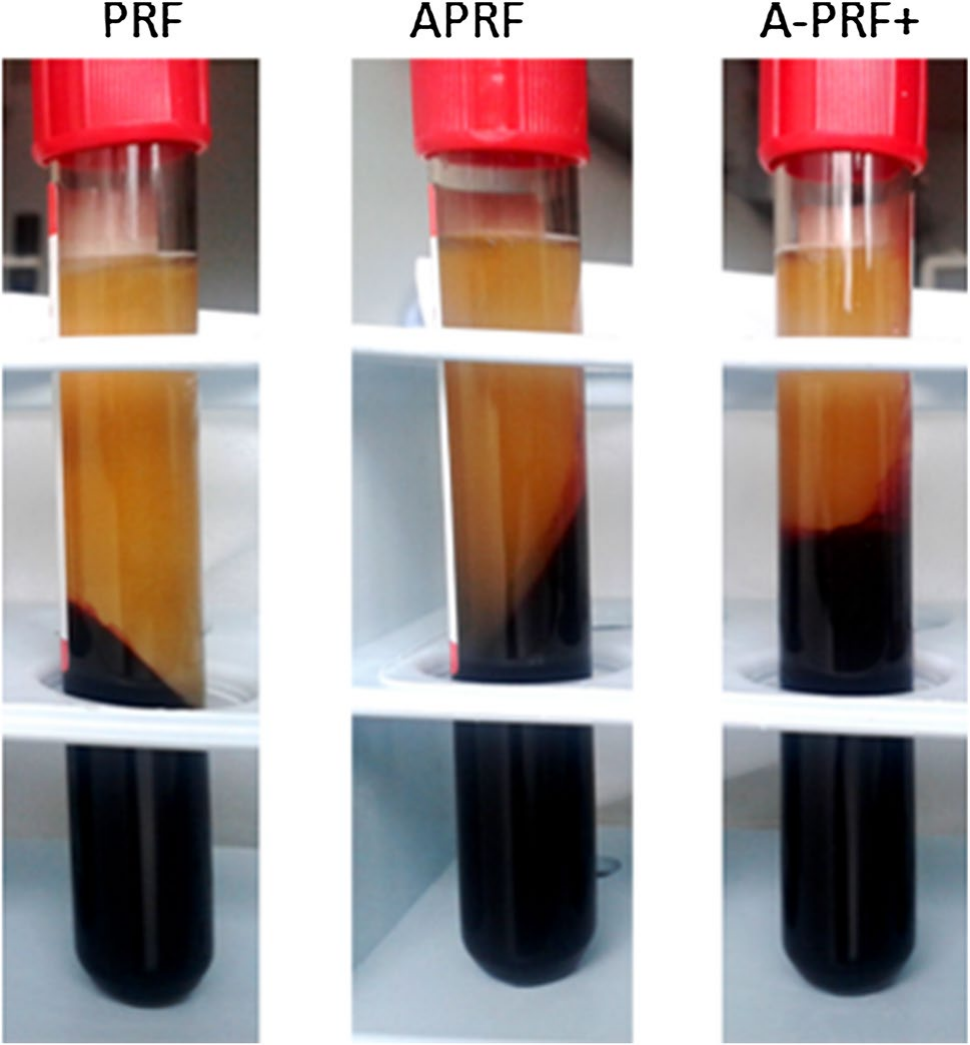
The PRF-based matrices immediately following centrifugation

Macroscopic observation demonstrated the formation of three slightly different clots. PRF formed a clot with a fibrin/red blood count (RBC) ratio of 1/1.66, and the clot length was measured as 3.5 cm. A-PRF showed a clot formation with a fibrin/red blood count (RBC) ratio of 1/2. Here the clot length was 3.5 cm. A-PRF+ had a fibrin/red blood count (RBC) ratio of 1/3 and a length of 2.5 cm (Fig. 1). Moreover, while separating the fibrin clot from the RBC, it was observed that in the case of PRF and A-PRF, the adhesion between the two sections, the fibrin clot and RBC, was stronger compared with A-PRF+. Accordingly, the A-PRF+ fibrin clot was much easier to separate.

### Growth factor release kinetics from the clots

The present study focused on the determination of the released growth factor kinetics of the three PRF-based matrices, PRF, A-PRF and A-PRF+. The growth factors VEGF, EGF and TGF-β1 were quantified for the released concentrations at each time point (6, 24, 48, 72 h, 7, and 10 days). Additionally, the accumulated growth factor quantities were calculated.

#### VEGF release

**Fig. 2:**
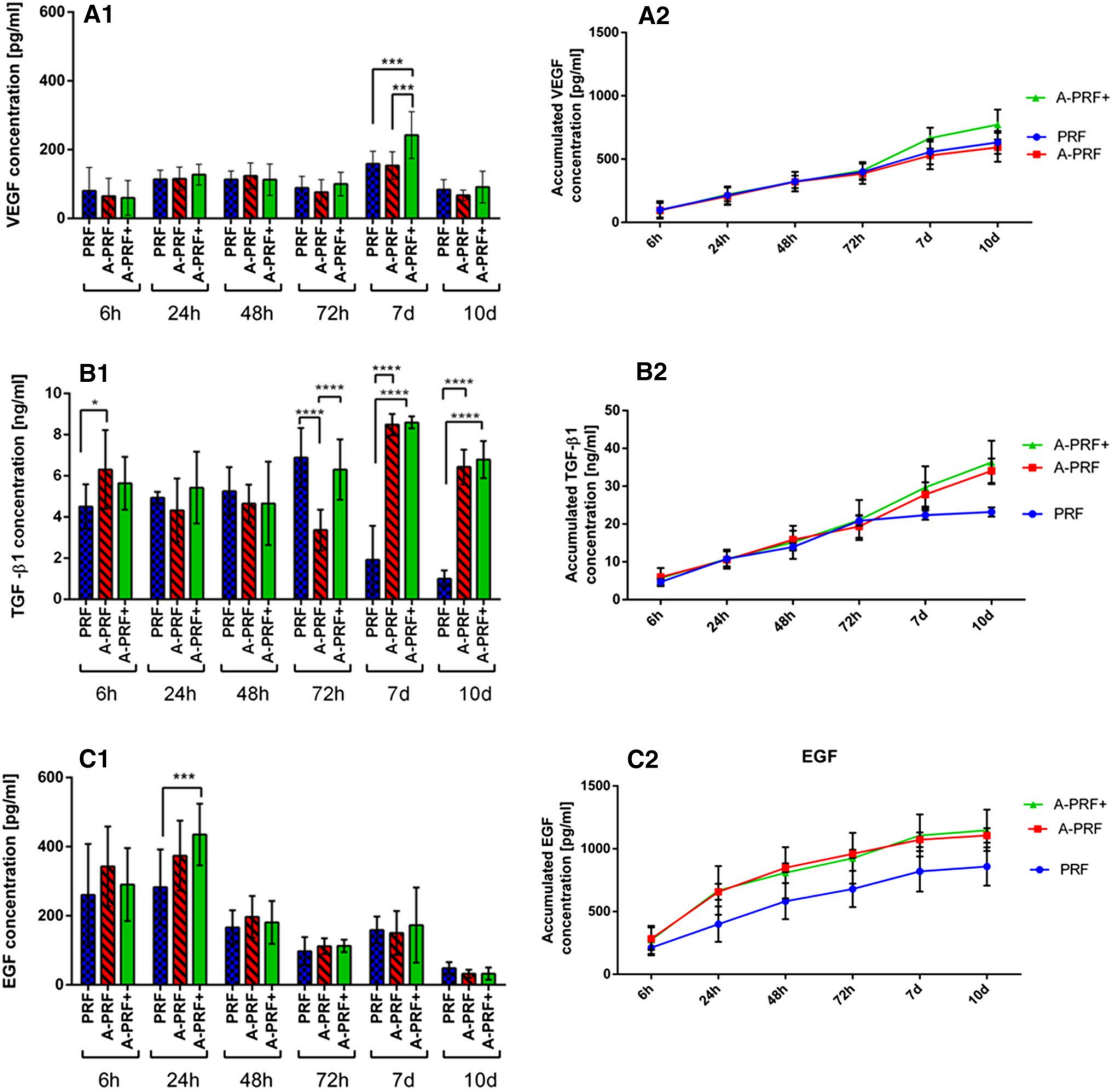
Statistical analysis of the growth factor releases by time points as the mean ± standard deviation for PRF, A-PRF and A-PRF+. **a1** VEGF, **b1** TGF-β1 release, **c1** EGF release, (*p < 0.05), (***p < 0.0005), (****p < 0.0001). Total accumulated growth factor concentration over 10 days. **a2** VEGF, **b2** TGF-β1, **c2** EGF

The general trend of the three evaluated groups at each time point was similar. The release of VEGF increased in the very early phase from 6 to 24 h in all groups. At 48 h, the growth factor release was comparable to the values at 24 h in all groups. From 48 to 72 h, a slight decrease in the release of VEGF was evidenced in all groups. From 72 h to day 7, a highly significant increase in all groups was observed (*p* < 0.0005) in an intra-individual comparison (data not shown). During the 4 days of cultivation between 72 h and day 7, the highest released concentration of VEGF over the study time was measured. Here, A-PRF+ showed the highest concentration when compared with PRF and A-PRF (PRF = 158.5 ± 36.6 pg/ml; A-PRF = 153.6 ± 40.1 pg/ml; A-PRF+ = 242.35 ± 67.9 pg/ml), which was statistically highly significant when compared to PRF and A-PRF (*p* < 0.0005). By contrast, A-PRF showed no statistically significant difference compared to PRF. From day 7 to day 10, all groups showed a decrease in the release of VEGF. This decrease was intra-individually statistically highly significant compared with day 7 (data not shown). Furthermore, after 10 days, A-PRF+ showed the highest VEGF release (PRF = 83.7 ± 28.81 pg/ml; A-PRF = 64.84 ± 15.7 pg/ml; A-PRF+ = 95.5 ± 44.7 pg/ml). At this time point, no significant difference could be identified among the groups (Fig. 2a1).

Concerning the accumulated VEGF concentration, a general trend was also evidenced by a continuous increase in the released VEGF over the study time. In the early phase (6–72 h), the release of VEGF increased in all groups, whereas the groups’ concentrations were quite similar. Moreover, in the late study period (72 h–10 days), a similar tendency was observed in all groups. However, A-PRF+ released the highest concentration on day 10 when compared with PRF and A-PRF (Table 1). This difference was highly significant when comparing A-PRF+ to A-PRF (***p < 0.0005) and significant comparing A-PRF+ to PRF (**p < 0.005) at this time point (Fig. 2 a2).

**Table 1:** Accumulated growth factor concentration of PRF, A-PRF and A-PRF+ at day 10 as the mean ± standard deviation. Statistical analysis of A-PRF and A-PRF+ compared with PRF (*p < 0.05), (**p < 0.005), (***p < 0.0005), (****p < 0.0001)

Growth factor	PRF	A-PRF	A-PRF+
VEGF (pg/ml)	632.26 ± 90.58	593.15 ± 114.08	773.88 ± 117.66**
TGF β1 (ng/ml)	23.18 ± 1.22	34.081 ± 3.21****	36.29 ± 5.73****
EGF (pg/ml)	858.62 ± 152.90	1106 ± 57.74*	1147.07 ± 164.47**

#### TGF-β1 release

Various TGF-β1 release patterns were measured in PRF, A-PRF and A-PRF+. Within the PRF group, a slight increase was observed in the early study time (6–72 h) followed by a dramatic decrease in the late study time (72 h–10 days). At 72 h, PRF already showed the highest concentration over the study period. At this time point, PRF was significantly higher only when compared to A-PRF (*p* < 0.0001), whereas no significant difference was observed compared to A-PRF+ (Fig. 2b1).

The A-PRF group showed a high release value at the first time point (6 h) (PRF = 4.6 ± 1.0 ng/ml; A-PRF = 7.0 ± 1.4 ng/ml; A-PRF+ = 5.8 ± 1.4 ng/ml), the difference between A-PRF and PRF being statistically significant (*p* < 0.05). However, no statistically significant difference was detected regarding A-PRF+. This observation was followed by irregular behavior until 72 h and a significant increase at day 7, when the highest TGF-β1 release of A-PRF was observed. At this time point, A-PRF was significantly higher than PRF (*p* < 0.0001), whereas no significant difference was revealed for the A-PRF+ group.

A-PRF+ showed a mild decrease of the released TGF-β1 at the early study time (6–48 h). However, from 72 h to day 7, an increase in the released TGF-β1 was observed when the highest concentration of TGF-β1 release was reached in the case of A-PRF+. At day 7, a statistically highly significant difference was observed when compared with PRF (*p* < 0.0001), whereas no significant difference was observable compared to A-PRF (PRF = 1.9 ± 1.6 ng/ml; A-PRF = 8.5 ± 0.6 ng/ml; A-PRF+ = 8.6 ± 0.4 ng/ml). From day 7 to day 10, the release of TGF-β1 decreased in all groups. However, A-PRF showed significantly higher values when compared with PRF (*p* < 0.0001). Similarly, A-PRF+ revealed more growth factor release, which was highly significant when compared with PRF (*p* < 0.0001). No statistically significant difference was observed when comparing A-PRF and A-PRF+ at this time point (Fig. 2b1).

The accumulated concentration of TGF-β1 showed an increase in all groups at the early study time (6–72 h). However, at the late study time (72 h–10 days), the growth factor release differed among the various groups. PRF showed a more or less constant concentration of TGF-β1 after 72h, whereas in the case of A-PRF and A-PRF+, an increased TGF-β1 concentration was observed. These differences on day 10 were statistically significant when comparing A-PRF to PRF (*p* < 0.0001) and A-PRF+ to PRF (*p* < 0.0001); however, no statistically significant difference was detected when comparing A-PRF to A-PRF+ (Table 1) (Fig. 2b2).

#### EGF release

A general trend was observed in all three PRF-based matrices. The rate of the released EGF increased quite early in the study time (6–24 h) to reach the highest value in all groups at 24 h. At this time point, A-PRF+ showed the highest value of the released EGF when compared with PRF and A-PRF (PRF = 282.69 ± 109.09 pg/ml; A-PRF = 373.75 ± 101.25 pg/ml; A-PRF+ = 435.17 ± 89.29 pg/ml), the difference being statistically highly significant when comparing A-PRF+ to PRF (***p < 0.0005); no statistical significance was observed when comparing A-PRF to A-PRF+. Subsequently, a course change was observed when a strong reduction of the released EGF occurred in all examined groups until 72 h. After that, on day 7, a slight increase was observed in all groups. Here also, A-PRF+ was the highest (PRF = 148.28 ± 48.27 pg/ml; A-PRF = 138.70 ± 61.07 pg/ml; A-PRF+ = 173.50 ± 98.72 pg/ml) although no statistically significant difference was detectable. At the last evaluated time point on day 10, all groups showed a significant decrease in the released EGF compared with day 7 (data not shown). However, at this time point, no statistically significant differences were observed among the groups (Fig. 2c1).

The accumulated concentration of the released EGF also exhibited a general trend. All groups showed a similar curve progression in the form of increased EGF release over the study time. A-PRF and A-PRF+ also displayed similar values. Early in the study time, a remarkable increase in released EGF was evidenced in all groups. After 72 h, only a minor increase of the released EGF was observed toward the end of the study on day 10. At these time points (72 h–10 days), A-PRF and A-PRF+ showed statistically significantly higher release values when compared with PRF (A-PRF+ compared with PRF *p* < 0.005; A-PRF compared with PRF *p* < 0.05), whereas no statistically significant differences were revealed when comparing A-PRF to A-PRF+ (Table 1) (Fig. 2c2).

### Platelet distribution in the PRF-based matrices

**Fig. 3:**
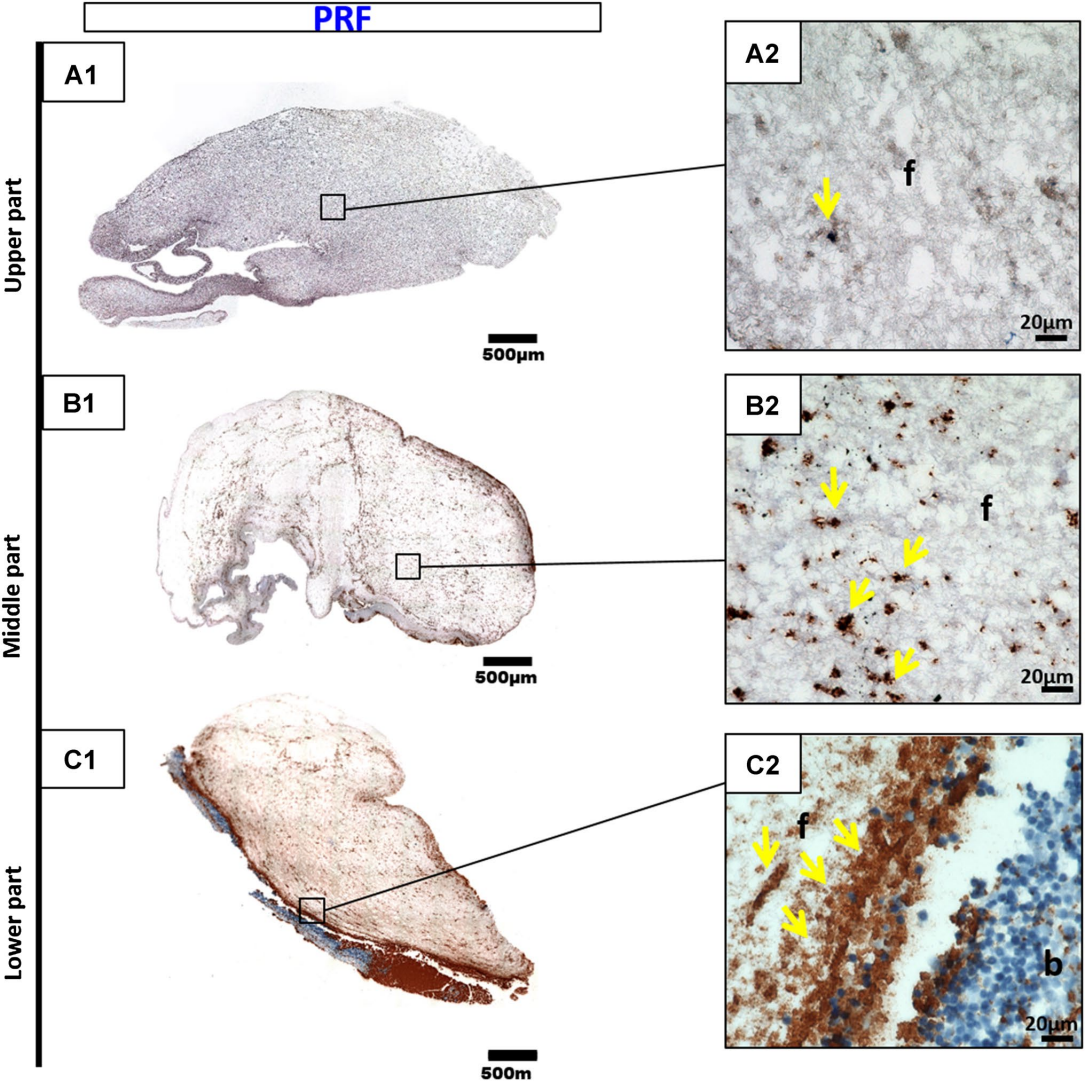
CD-61 immunohistochemical analysis of PRF according to the different regions. **a1, a2** upper portion; **b1, b2** middle portion; **c1, c2** lower portion (**a1, b1, c1** total scan sections; ×100 magnification, *scale bar* 500 µm). **a2, b2, c2** show the distribution pattern of platelets (*yellow arrows*) in higher magnification (*f* fibrin; *b* buffy coat; ×400 magnification; *scale bar* 20 µm)

**Fig. 4:**
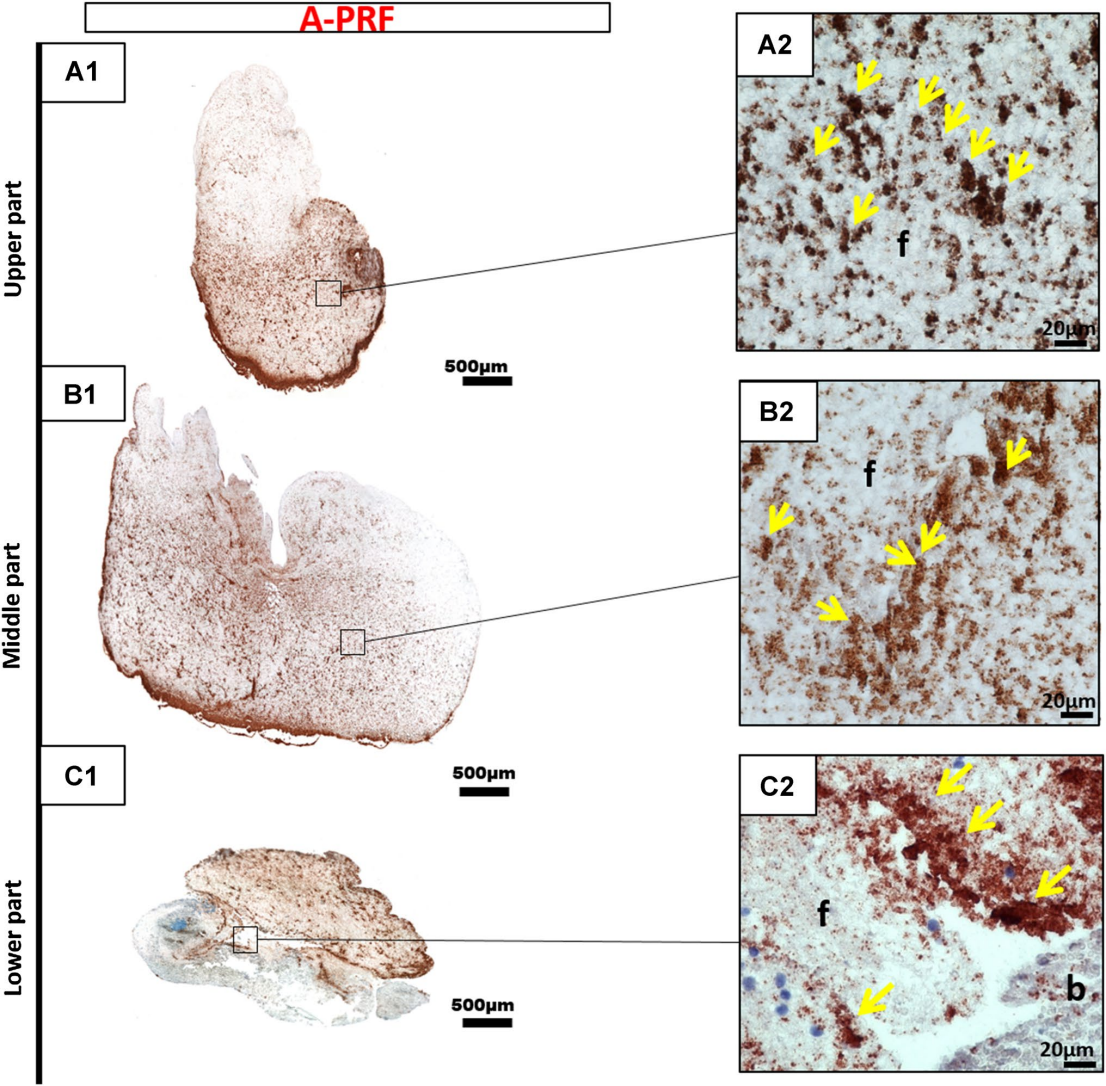
CD-61 immunohistochemical analysis of A-PRF according to the different regions. **a1, a2** upper portion; **b1, b2** middle portion; **c1, c2** lower portion (**a1, b1, c1** total scan sections; ×100 magnification, *scale bar* 500 µm). **a2, b2, c2** Show the distribution pattern of platelets (*yellow arrows*) in higher magnification (*f* fibrin; *b* buffy coat; ×400 magnification; *scale bar* 20 µm)

**Fig. 5:**
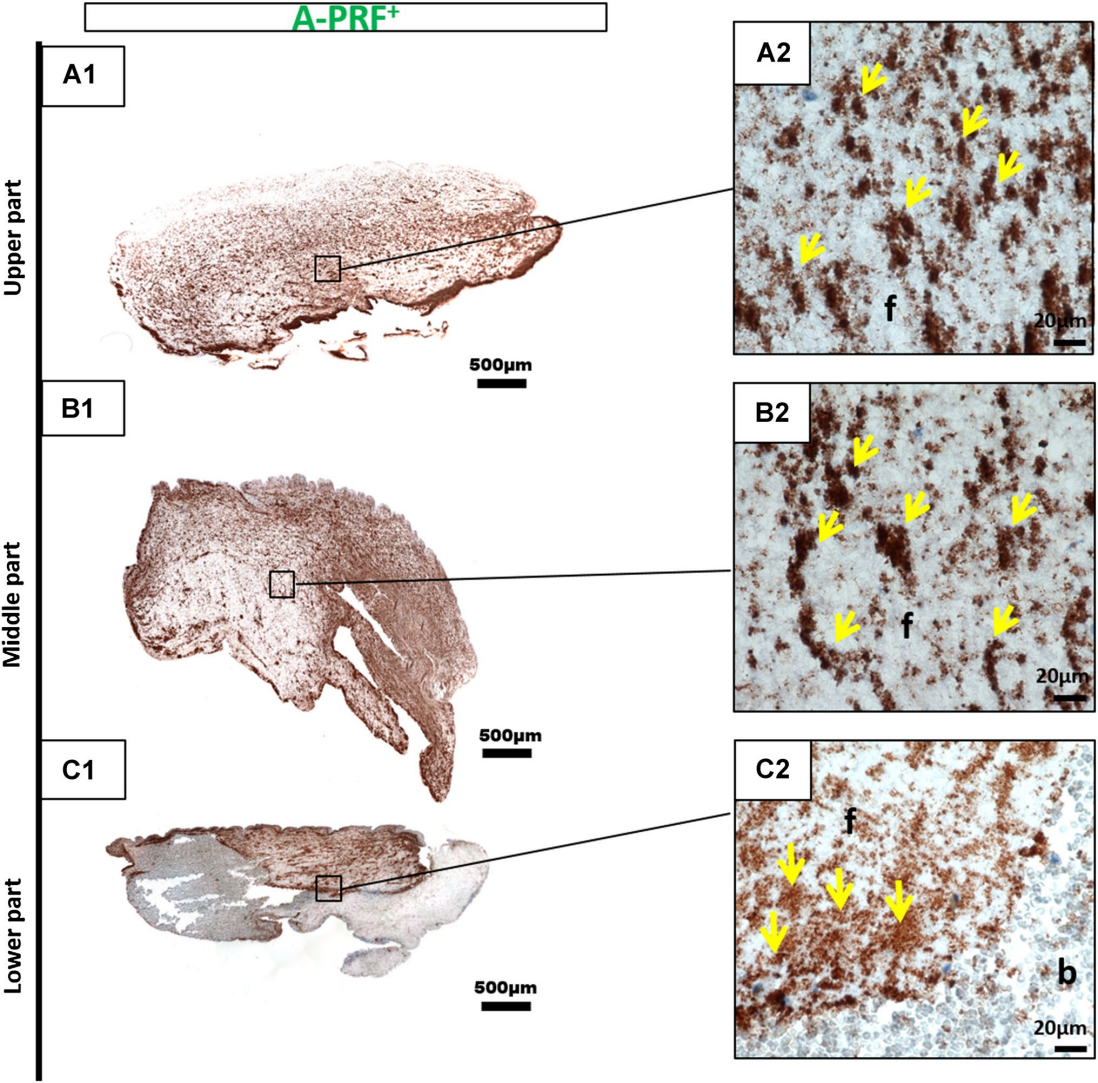
CD-61 immunohistochemical analysis of A-PRF+ according to the different regions. **a1, a2** upper portion; **b1, b2** middle portion; **c1, c2** lower portion (**a1, b1, c1** total scan sections; ×100 magnification, *scale bar* 500 µm). **a2, b2, c2** Show the distribution pattern of platelets (*yellow arrows*) in higher magnification (*f* fibrin; *b* buffy coat; ×400 magnification; *scale bar* 20 µm)

Immunohistochemical staining with CD-61 antibodies against platelets was conducted to determine the platelet distribution in cross sections of the three PRF-based matrices. The platelet distribution was evaluated with regard to the location in the clot. The platelets formed accumulations within all three clots. PRF, which was prepared with a high RCF, showed a different distribution pattern according to the localization. The upper and middle portions of the clot showed only a few platelets, whereas the majority of platelets were distributed in the lower portion of PRF (Fig. 3). By contrast, A-PRF, which was prepared with a reduced RCF, presented a different distribution pattern. Platelets were dispersed all over the clot (Fig. 4). A-PRF+ with a reduced RCF and a reduced centrifugation time also displayed an even platelet distribution pattern in the various locations within the clot (Fig. 5).

## Discussion

This study presents the potential of PRF-based matrices (PRF, A-PRF and A-PRF+) for growth factor release as a modest contribution to ongoing discussions regarding the preparation of PRF-based matrices as biological scaffolds and a natural growth factor release system, which is derived from autologous blood. The results revealed continuous growth factor release of VEGF, TGF-β1 and EGF over the study time. However, statistically significant differences among the various preparation protocols, PRF, A-PRF and A-PRF+, were demonstrated.

One of the most potent angiogenesis-stimulating growth factors is VEGF. A-PRF+ released significantly more VEGF than PRF and A-PRF on day 7. Moreover, the accumulated release of VEGF on day 10 was significantly higher in A-PRF+ than in PRF and A-PRF. However, no statistical significance was detected when evaluating A-PRF and PRF. These outcomes are quite likely related to the specific fibrin structure and cellular distribution pattern of A-PRF+. VEGF plays a crucial role in wound healing and tissue regeneration to promote vascularization and new vessel formation [17]. Additionally, previous studies have demonstrated that the sustained release of VEGF promotes epithelialization and enhances collagen tissue deposition in a skin wound healing model in mice [18]. Thus, the sustained and enhanced VEGF release of A-PRF+ could lead to more benefits in regeneration and vascularization and thus provide a nutrient supply to support wound healing and improve the biomaterial-guided regeneration pattern.

The release of TGF-β1 in A-PRF and A-PRF+ indicated the maximal release values on days 7 and 10, which were significantly higher when comparing A-PRF to PRF and A-PRF+ to PRF. However, no statistically significant difference between the TGF-β1 release of A-PRF and A-PRF+ was identified. On day 10, the accumulated TGF-β1 concentration was significantly higher in the A-PRF and A-PRF+ groups than in the PRF group. By contrast, A-PRF and A-PRF+ revealed no statistically significant difference in this case. TGF-β 1 is essential for wound healing [19]. Chronic wounds were observed to have a decreased expression of TGF-β receptors [20]. Thus, PRF matrices with an enhanced release of TGF-β1, as was the case for A-PRF and A-PRF+, could have a major influence on wound healing as a catalyzer of wound repair stages. In addition, this growth factor is known to stimulate fibroblast migration, enhance collagen synthesis and promote angiogenesis [21, 22]. All of the latter characteristics are essential in the biomaterial-based regeneration process. Hence, PRF-based matrices as an additional autologous dose of inflammatory cells and growth factor could be promising in the field of guided bone and tissue regeneration (GTR and GBR), in which biomaterials should provide a scaffold and support the regeneration process in the defect area.

The release of EGF was generally higher in the A-PRF and A-PRF+ groups when compared with PRF. Statistically highly significant differences were detected when comparing A-PRF+ with PRF after 24 h, whereas no significant difference was observed between A-PRF+ and A-PRF. The accumulated EGF release showed significantly higher rates in the case of A-PRF and A-PRF+ compared with PRF at most time points, particularly on day 10. EGF has previously been described as promoting cell growth [21], enhancing keratinocyte migration [23], inhibiting apoptosis under hypoxic conditions [24], and supporting re-epithelization and skin healing [25, 26]. Additionally, EGF supports the healing process of chronic wounds [27], non-healing chronic wounds and ulcers, which are, for example, observed in diabetic patients known to lack the necessary growth factors to maintain the healing process [28, 29]. Thus, such patient groups may benefit from the application of PRF matrices as an autologous drug delivery system. Moreover, immunohistochemical evaluation indicated an equal distribution pattern of platelets in all clot regions in the case of A-PRF and A-PRF+, whereas in PRF, the majority of the platelets were located in the lower portion of the clot. These findings may be related to the LSCC (low speed centrifugation concept), indicating that reducing the applied RCF increases the number inflammatory cells and platelets as well as the growth factor release within the PRF-based matrices [15]. Because the centrifugation process depends on cell weight and density, a higher RCF may be the reason for the sedimentation of the majority of the platelets to the lower portion of the clot according to their density and size, as observed in PRF. Decreasing the RCF allows the platelets to become separated from the red blood cell phase and become equally distributed within the fibrin network. The effectiveness of PRF clots with low platelet counts and uneven platelet distribution may have less influence on clinical outcomes than clots with evenly distributed and enhanced platelet numbers because the applied clot could have uneven biological activity and thus a reduced growth factor release, as indicated in the present study. However, comparative clinical studies are necessary to evaluate the advanced PRF matrices presented here to demonstrate the extent to which the improved structure, even cellular distribution and enhanced growth factor release may affect clinical outcomes.

These observations highlight the influence of RCF reduction, i.e., from PRF (708 g) to A-PRF and A-PRF+ (208 g) on platelet distribution, thereby correlating with the previously demonstrated automated cell counting that indicated significantly more platelets in PRF matrices prepared with low RCF than with high RCF application [15]. A previous *ex vivo* immunohistochemical study demonstrated the distribution pattern in PRF and A-PRF, which included, in addition to platelets, a wide range of inflammatory cells that physiologically exist within the peripheral blood, such as leukocytes, including neutrophils and monocytes as well as lymphocytes [5]. However, further immunohistochemical studies are required to determine the distribution pattern of the included leukocytes and their subgroups, particularly in A-PRF+. These cells, particularly platelets and neutrophilic granulocytes, contribute to neoangiogenesis and VEGF release [30, 31]. In addition, platelets are the primary secretory cells of EGF and TGF-β1 [32]; thus, their presence within the PRF-based matrices is a possible explanation for the observed growth factor release. These cells are essential for wound healing and tissue regeneration [33, 34]. In the present study, release kinetics displayed an increased growth factor release over the study time and a maximum at day 7 in the case of VEGF and TGF-β1 as well as an increased growth factor release at 24 h in the case of EGF. Based on the growth factor and release kinetics demonstrated here, one may assume that the growth factor release pattern within the various PRF-based matrices is an active release from living cells within the different PRF clots, which most likely experienced apoptosis during the study period if 10 days reflects the reduction in growth factor release at day 10 compared with day 7 in all groups and growth factors.

Additionally, leukocytes and platelet interaction via cellular cross talk have been described in bone regeneration [9]. In this context, the high regeneration potential of advanced PRF-based matrices could be beneficial in various clinical applications, such as enhancing the regeneration pattern of biomaterials in terms of GTR and GBR. Moreover, autologous biologizing biomaterials using PRF-based matrices may improve the regeneration pattern in large-sized, soft and bony defects to catalyze wound healing and regeneration. Ongoing clinical observations in oral- and maxillofacial surgery have demonstrated that various bony defects within the jaw or head can be regenerated by different clot numbers according to the defect size. Thus, molar sockets are treated with 2–3 clots, whereas larger bony head defects are treated with up to 6 clots. Based on these observations, PRF-based matrices could be a beneficial tool to improve the regeneration of soft and bony defects after orthopedic or trauma surgery. The present study demonstrates that the application of the LSCC (low speed centrifugation concept), by decreasing the RCF from PRF toward A-PRF and A-PRF+, results in a significantly higher release of VEGF, TGF-β1 and EGF. Notably, the accumulated release over 10 days of TGF-β1 and EGF supports the relation between the reduction of RCF and the growth factor release. Hence, A-PRF+ and A-PRF, which were prepared with the same RCF, displayed comparable results that were significantly higher than PRF, which was prepared with more than three times higher RCF. These observations emphasize the fact that the application of the LSCC is valuable in modifying and optimizing solid PRF-based matrices. However, the manipulation of the centrifugation time appeared to influence only certain growth factors, as shown in the case of A-PRF+. The accumulated VEGF release on day 10 showed a significantly higher rate in the group of A-PRF+ compared with A-PRF and PRF. It may be that the application of a low RCF but a longer centrifugation time, as demonstrated in the case of A-PRF, affected the VEGF release capacity, whereas the application of a low RCF and slightly decreased centrifugation time, as in A-PRF+, resulted in a significantly higher VEGF release. Another plausible explanation may be that the specific fibrin clot composition of A-PRF+ allows a highly increased VEGF release and thus a higher accumulated VEGF release on day 10. These data accentuate the fact that the various growth factor concentrations may be a consequence of the various total cell concentrations within the PRF-based matrices.

The various release profiles of the evaluated PRF-based matrices may also be a consequence of the different growth factor binding affinities to fibrin. It has been demonstrated that growth factors, such as VEGF, have a high affinity to bind to fibrinogen and fibrin so that those factors are released in a sustained manner [35]. This information is reflected in the present results by showing significantly enhanced VEGF release on day 7 in the case of A-PRF+. By contrast, EGF is released in a high concentration level at the very early time point of 24 h. One explanation for this observation may be the low binding affinity of EGF to fibrin and fibrinogen [36]. Another factor may be the structure of the PRF-based matrices. A-PRF and A-PRF+ exhibit a more porous structure than the densely structured PRF [5]. The physical properties of the clot and the specific fibrin structure related to the manufacturing protocol [5] may also influence the binding affinity and the sustained release of the various growth factors. It is possible that a more porous structure, as shown in A-PRF and A-PRF+, is one reason for an enhanced growth factor release [5]. Thus, it remains questionable whether the growth factor release is related to the specific physical properties of the fibrin network or to the included inflammatory cells and platelets, or perhaps a combination of both. Therefore, further study is required to understand this specific complex system.

The release kinetics of growth factors in the PRF-based matrices have previously been reported in several studies [37, 38]. Direct comparisons of these studies are limited because of the various preparation protocols in terms of RCF, centrifugation time, blood volume and the techniques used to generate the PRF-based matrices. However, one in vitro study analyzed the growth factor release in PRF-based matrices compared with PRP [39]. Correlations were demonstrated in the case of the accumulated TGF-β1 and EGF, for which both studies presented a significantly higher growth factor release in PRF matrices prepared with a low RCF application compared with PRF matrices with high RCF exposure. This accentuates the fact that reduction of the RCF enhances the release of these growth factors. Notably, the later study also showed that PRP released higher growth factor concentrations (EGF, VEGF and TGF-β1) at the very early time points, whereas PRF-based matrices showed a continuous and higher growth factor concentration over a period of 10 days [39]. Moreover, this group demonstrated further evaluation of the growth factors in PRF, A-PRF and A-PRF+ [40]. The results of the accumulated growth factor release on day 10 are consistent with the present findings with regard to A-PRF+ concerning TGF β1 and EGF. Both studies presented a significantly higher release of these growth factors within A-PRF+ when compared with PRF. By contrast to Kobayashi et al. (2016), the present study reveals no significant differences between A-PRF and A-PRF+ with regard to TGF β1 and EGF. Additionally, the present outcomes indicate significantly higher accumulated VEGF release on day 10 in the group of A-PRF+ compared with A-PRF and PRF, whereas Kobayashi et al. (2016) showed no statistically significant differences between the examined groups on day 10. At this point, it must be stressed that the two studies were of different designs. Kobayashi et al. (2016) evaluated different time points from the time points investigated in the present study. In addition, Kobayashi et al. (2016) used a shaking incubator before performing the ELISA evaluation, whereas our group incubated the PRF-based matrices without further manipulation, which can also be a reason for the discrepancies revealed in the results. It is evident that detection of the specific growth factors is dependent on the specific methods employed. Thus, further studies in this field are necessary to develop and evaluate PRF-based matrices generated according to LSCC.

The present experimental design regarding the preparation and cultivation of PRF-based matrices may offer advantages because the PRF clots were not compressed or manipulated but nevertheless yielded the large amount of growth factors in the PRF clot. Furthermore, the clots were incubated in a cell culture environment to provide adequate gas exchange and optimal conditions for cells. The primary limitation of this study is the in vitro system issue. A comparison with clinical results is difficult because of the discrepancy of comparing the physiological environment in vivo. Thus, the cellular crosstalk and enzymatic degradation of the fibrin network would be different in vivo. Further in vivo studies are required to determine the influence of the growth factors on the regeneration pattern of PRF-based matrices, particularly those matrices that are prepared according to the LSCC. This is necessary to identify out whether the observed inflammatory cell and growth factor enhancement will contribute to an improved regeneration potential in vivo. Moreover, the optimal release of growth factors required in wound healing and regeneration processes remains unclear, as is whether enhancing the amount released will indeed lead to improved performance. Thus, controlled clinical studies are essential to evaluate the regeneration potential of A-PRF and A-PRF+ and to establish the extent to which homogeneously distributed platelets and an enhanced growth factor release in addition to the porous structure will contribute to improved wound healing.

Less is known regarding the interaction of the PRF-based matrices with biomaterials with a view to improving biomaterial-based regeneration. In addition, little attention has been focused on the composition of PRF-based matrices obtained from patients undergoing pharmacologic treatments and whether the growth factor release will be influenced by medication. In addition, the regeneration potential of the PRF-based matrices may also be related to the age of the donor. Therefore, it may be that as the age of donors increases, less growth factor is released and vice versa. If this scenario is true, PRF-based matrices with enhanced growth factor release may be beneficial in these specific cases. In this respect, the determination of mononuclear cell growth in PRF and penetration into the PRF-based matrices as a simulation of the regeneration process in vitro would be of interest in understanding the role of PRF-based matrices in biomaterials and tissue engineering. Hence, further studies of the PRF-based matrices as a complex system that influences cell growth and differentiation and provides a growth factor reservoir remain necessary.

Additionally, the current PRF-based matrices were prepared according to specific protocols with a defined amount of blood. However, it would be interesting to determine how increasing or decreasing the blood volume influences the composition of the prepared PRF-based matrices, their regenerative potential and their growth factor release. These questions are current investigation topics of our research group as we seek to enhance wound healing and tissue regeneration to decrease patient morbidity. Hence, the outcomes of this study could provide new clinical approaches in tissue and bone regeneration in terms of a combination of biomaterials with PRF-based matrices. Nevertheless, further studies, particularly clinical studies, are required to develop optimized, standardized and tailored preparation protocols for various clinical applications and to demonstrate their advantages now and in the future.

## Conclusion

The present study demonstrates the influence of RCF reduction on the growth factor release and platelet distribution in solid PRF-based matrices. A-PRF+, prepared with a reduced RCF, displayed significantly higher VEGF concentration over the study period of 10 days than A-PRF and PRF, which exhibited no statistically significant difference. EGF and TGF-β1 were comparable in A-PRF and A-PRF+, which were significantly higher than PRF. Additionally, the platelet distribution pattern appeared to be equivalent in all regions concerning A-PRF and A-PRF+, whereas PRF showed the largest accumulation of platelets in the lower portion of the clot. Long-term, sustained and slow release of growth factors from all of the PRF groups may support cell migration and cell proliferation as well as offer advantages in the wound healing process. However, the significantly enhanced release in A-PRF and A-PRF+ may render these matrices superior to PRF in specific clinical indications. These promising findings offer an excellent handling efficiency and new approaches to the clinical application of wound healing as well as soft and bone tissue regeneration. Nevertheless, further clinical studies must demonstrate the extent to which the application of LSCC to generate A-PRF and A-PRF+ will benefit clinical outcomes.

## Electronic supplementary material

Below is the link to the electronic supplementary material.

Supplementary material 1 (DOCX 13 KB)
