# Supplementary material for: Reduction of relative centrifugal forces increases growth factor release within solid platelet-rich-fibrin (PRF)-based matrices: a proof of concept of LSCC (low speed centrifugation concept)
Source: Eur J Trauma Emerg Surg. 2017 Mar 21;45(3):467–79. doi: 10.1007/s00068-017-0785-7 (PMC6579868; doi:10.1007/s00068-017-0785-7)
Supplement: Supplementary file 1 — Supplementary material 1 (DOCX 13 KB) [file 68_2017_785_MOESM1_ESM.docx]

# Dear Prof. Marzi, Editor *of European Journal of Trauma and Emergency Surgery*,

Frankfurt, February 15, 2017

We are submitting for your consideration the revision of our manuscript entitled

**“Reduction of relative centrifugal forces increases growth factor release within solid platelet-rich-fibrin (PRF)-based matrices: A proof of concept of LSCC (Low Speed Centrifugation Concept)“**

**”**

Dear Prof. Marzi,

Thanks to you, and the reviewer, for reviewing our manuscript. The manuscript has undergone some revisions concerning the noted criticisms. Please find attached a list of the changes with regard to the reviewer comments. I am thankful for the reviewers’ comments, which definitely helped to improve the manuscript.

Best regards,

Shahram Ghanaati
